# Hidradenocarcinoma^☆^^☆☆^

**DOI:** 10.1016/j.abd.2020.03.023

**Published:** 2021-02-05

**Authors:** Andrey Amorim de Lima, Monica Santos, Patricia Motta de Morais, Carlos Alberto Chirano Rodrigues

**Affiliations:** a Teaching and Research Department, Fundação de Dermatologia Tropical e Venereologia Alfredo da Matta, Manaus, AM, Brazil; b Department of Dermatopathology, Fundação de Dermatologia Tropical e Venereologia Alfredo da Matta, Manaus, AM, Brazil; c Department of Dermatological Surgery, Fundação de Dermatologia Tropical e Venereologia Alfredo da Matta, Manaus, AM, Brazil

*Dear editor,*

Cutaneous hidradenocarcinoma is a rare malignant neoplasm, originating from the eccrine sweat glands, and corresponding to 6% of the eccrine malignant tumors. It presents as a solitary, asymptomatic, slow-growing skin lesion, and may take an aggressive clinical course, with lymph node involvement and distant metastases.^1^, ^2^

Classically, it is a carcinoma reported with *de novo* onset, with no signs of previous hidradenoma. It most often affects patients from the fourth decade of life onwards.^3^ Classically, the head and neck are the most affected sites; however, it has been reported in several locations.^4^

In this study, the authors report the case of a 42-year-old female patient who sought care during the National Skin Cancer Campaign complaining of a nine-month-old lesion on the buttock. Dermatological examination evidenced the presence of an erythematous, hardened, asymptomatic tumor, of approximately 5 cm, located in the lateral region of the right gluteus with nine months of evolution (Fig. 1). She denied any trauma or previous injuries at the site.

**Figure 1:**
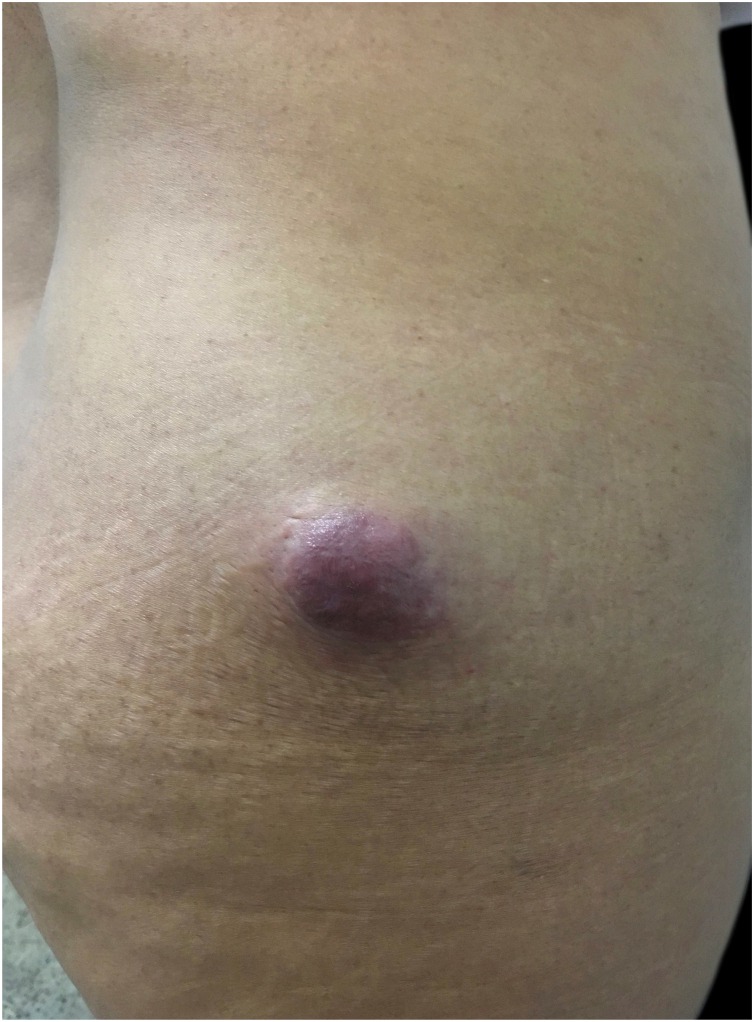
Asymptomatic, hardened, erythematous tumor, of approximately 5 cm, located in the lateral region of the right gluteus.

In view of the clinical picture, the hypotheses of dermatofibrosarcoma and proximal epitheloid sarcoma were raised. A 5-mm punch biopsy was performed for histopathological examination which revealed nests and cords of cuboidal cells in the deep dermis, with eosinophilic cytoplasm, mildly pleomorphic and hyperchromatic nuclei, and formation of tubular structures and ductal lumens, arranged in a myxoid and collagenous stroma, compatible with a skin adnexal tumor (Fig. 2).

**Figure 2:**
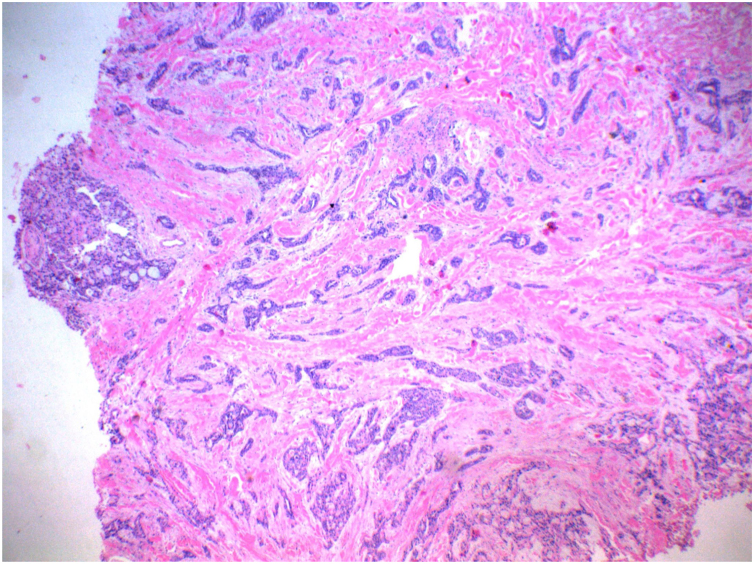
Presence, in the deep dermis, of nests and cords of cuboid cells, with eosinophilic cytoplasm, mildly pleomorphic and hyperchromatic, forming tubular structures and ductal lumens, arranged in a myxoid and collagenous stroma, compatible with adnexal skin tumor (Hematoxylin & eosin, ×40).

The patient returned two months after the biopsy; an increase in the size of the lesion and elimination of continuous hyaline secretion were observed. Spindle-shaped excision was performed, with a safety margin of 2 cm, without recurrence of the lesion after 12 months of follow-up. The immunohistochemical analysis of the excised lesion was positive for cytokeratins of 40, 48, 50, and 50.6 kDa (AE1/AE3), p63 protein (DAK-p63), epithelial tumor glycoprotein (BerEp4), and carcinoembryonic antigen – CEA (polyclonal). Partial positivity was observed for epithelial membrane antigen – EMA (E29). Histopathological and immunohistochemical findings favored the diagnosis of hidradenocarcinoma. Tumor blood markers, such as lactic dehydrogenase (DHL), alpha-fetoprotein, and CEA, were normal. Computed tomography (CT) of the abdomen and pelvis without contrast showed the presence of an oval mass with defined contours and margins in the right iliac fossa (ipsilateral to the excised tumor) adjacent to the sartorius muscle, measuring about 4.2 × 2.2 cm, heterogeneous, with areas of soft tissue density (32 uH) and interspaced slightly hypodense areas, with apparently non-specific liquid density. No abnormalities were observed in the abdominal and pelvic organs.

The patient was referred to an oncologic center. Seven months after the first approach, she underwent another surgery, performed by a surgical oncologist, to remove the oval mass in the right iliac fossa identified by CT of the pelvis. The histopathological study showed malignant neoplasia infiltration of the lymph node, consisting of the proliferation of cells with clear eosinophilic cytoplasm, vesicular nucleus, and evident nucleolus, with foci of ductal differentiation and solid areas, with frequent necrosis and numerous mitoses. The immunohistochemical panel of the affected lymph node showed expression of p63 protein, in addition to EMA and cytokeratin in the ductal areas, favoring the diagnosis of metastatic hidradenocarcinoma (Fig. 3). One year after diagnosis, the patient is being followed-up at the state oncology reference service, with no signs of local recurrence.

**Figure 3:**
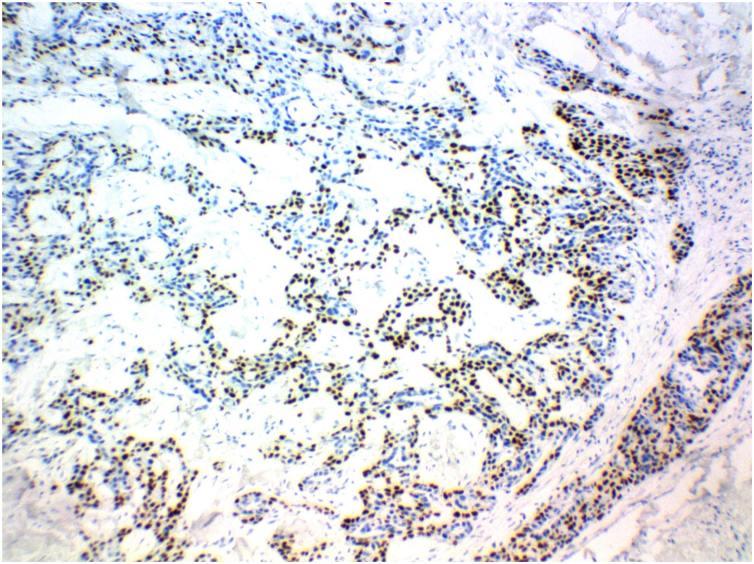
Immunohistochemical expression of p63 protein, favoring the diagnosis of metastatic hidradenocarcinoma (P63, ×100).

The diagnosis of hidradenocarcinoma is fundamentally based on histopathological and immunohistochemical findings. A panel positive for p63, CK15 and D2-40 is observed preferentially in primary adnexal tumors, while the negativity for these markers is found preferentially in skin metastases.^5^ In this report, the finding of immunoreactivity with p63 suggests that it was a primary adnexal neoplasia. Local recurrence of hidradenocarcinoma is observed in more than 50% of cases, and distant metastases are observed in approximately 60% of cases, mainly in lymph nodes, lungs, and bones, which is why it is necessary to keep following-up these patients and screening for metastases. The differential diagnosis is challenging because the lesion looks benign and similar to other skin tumors. Hidradenoma (benign variant), lipoma, hemangioma, lymphangioma, squamous cell carcinoma, basal cell carcinoma, malignant melanoma, dermatofibrosarcoma, and other benign and malignant adnexal tumors, in addition to metastatic tumors for the skin, are reported as differential diagnoses. The treatment of choice is complete surgical excision of the lesion with a wide safety margin. Due to the high local recurrence and regional lymph node involvement, surgery with a wide safety margin, not specified in the literature, and resection of regional lymph nodes followed by postoperative radiotherapy appear to be the appropriate initial therapy.^5^ It is important to highlight the role of the National Cancer Campaign carried out by the Brazilian Society of Dermatology, where the patient was first seen.

## Financial support

None declared.

## Authors’ contributions

Andrey Amorim de Lima: Drafting and editing of the manuscript; critical review of the manuscript.

Monica Santos: Approval of the final version of the manuscript; design and planning of the study; drafting and editing of the manuscript; intellectual participation in propaedeutic and/or therapeutic conduct of the studied cases; critical review of the literature; critical review of the manuscript.

Patricia Motta de Morais: Intellectual participation in propaedeutic and/or therapeutic conduct of the studied cases; critical review of the literature.

Carlos Alberto Chirano Rodrigues: Intellectual participation in propaedeutic and/or therapeutic conduct of the studied cases.

## Conflicts of Interest

None declared.
